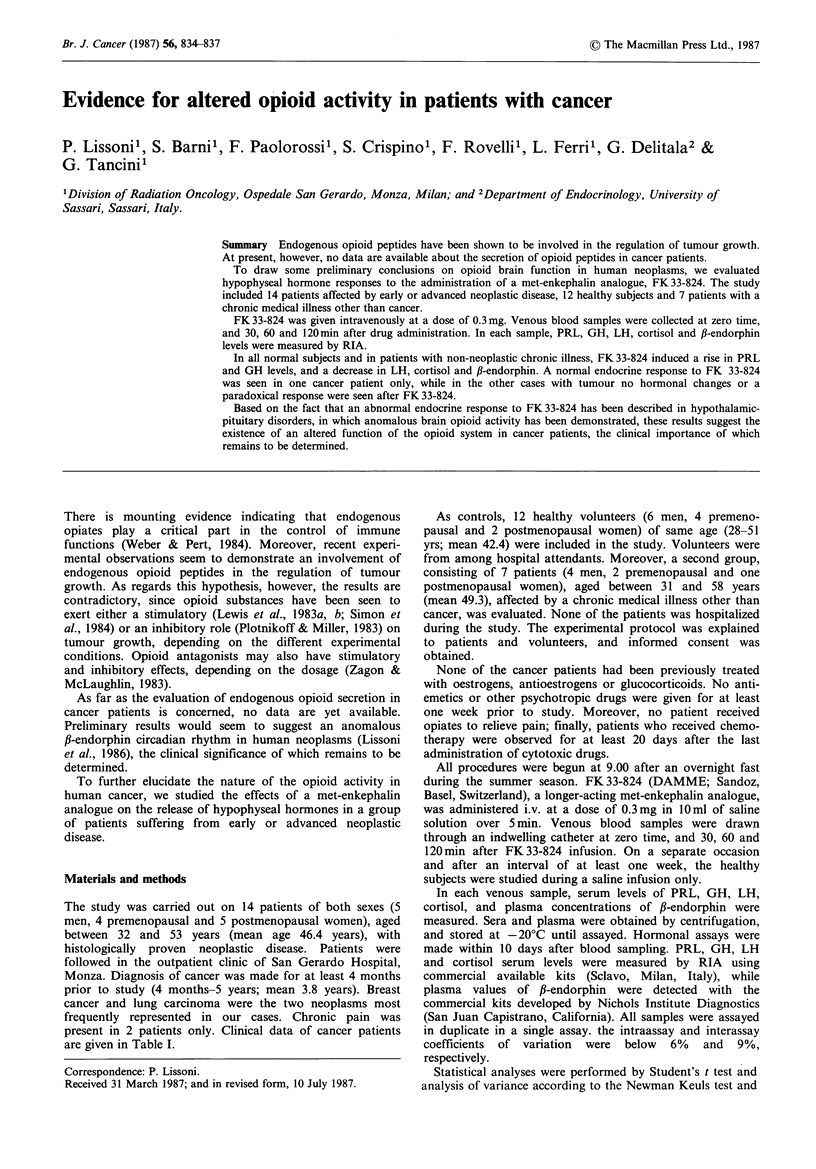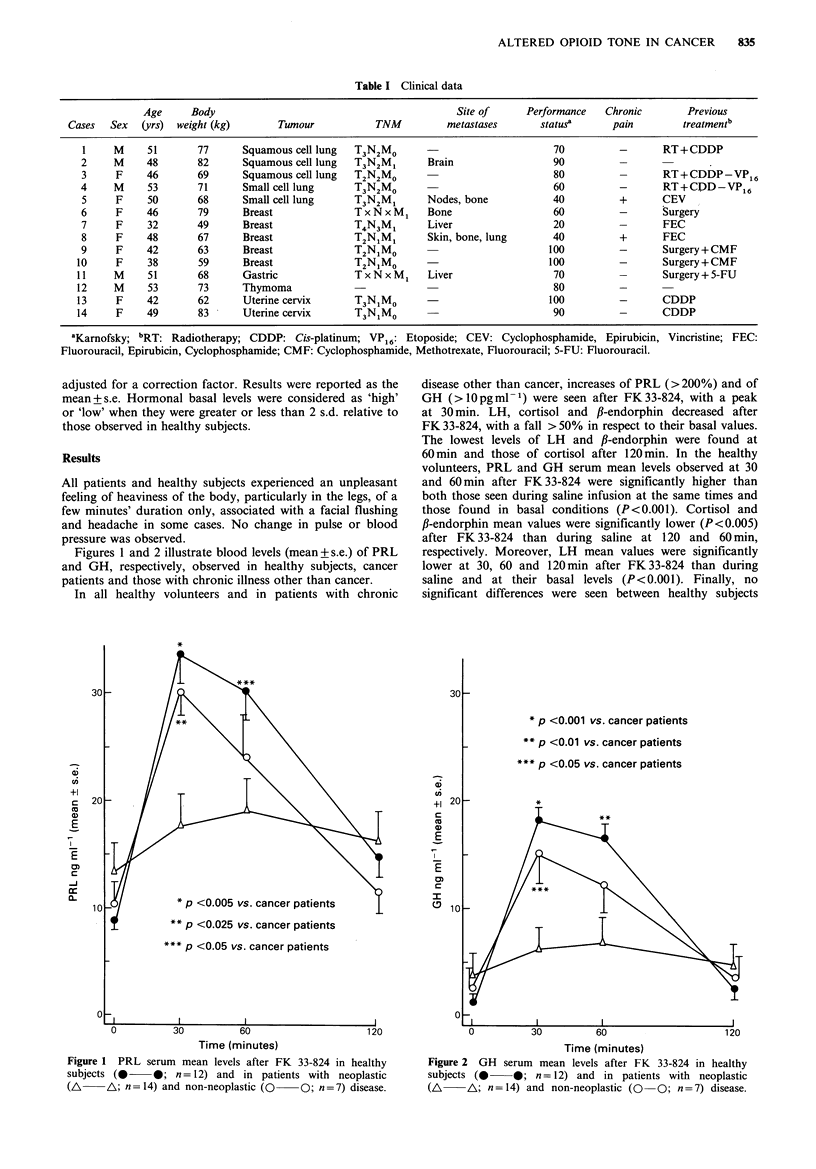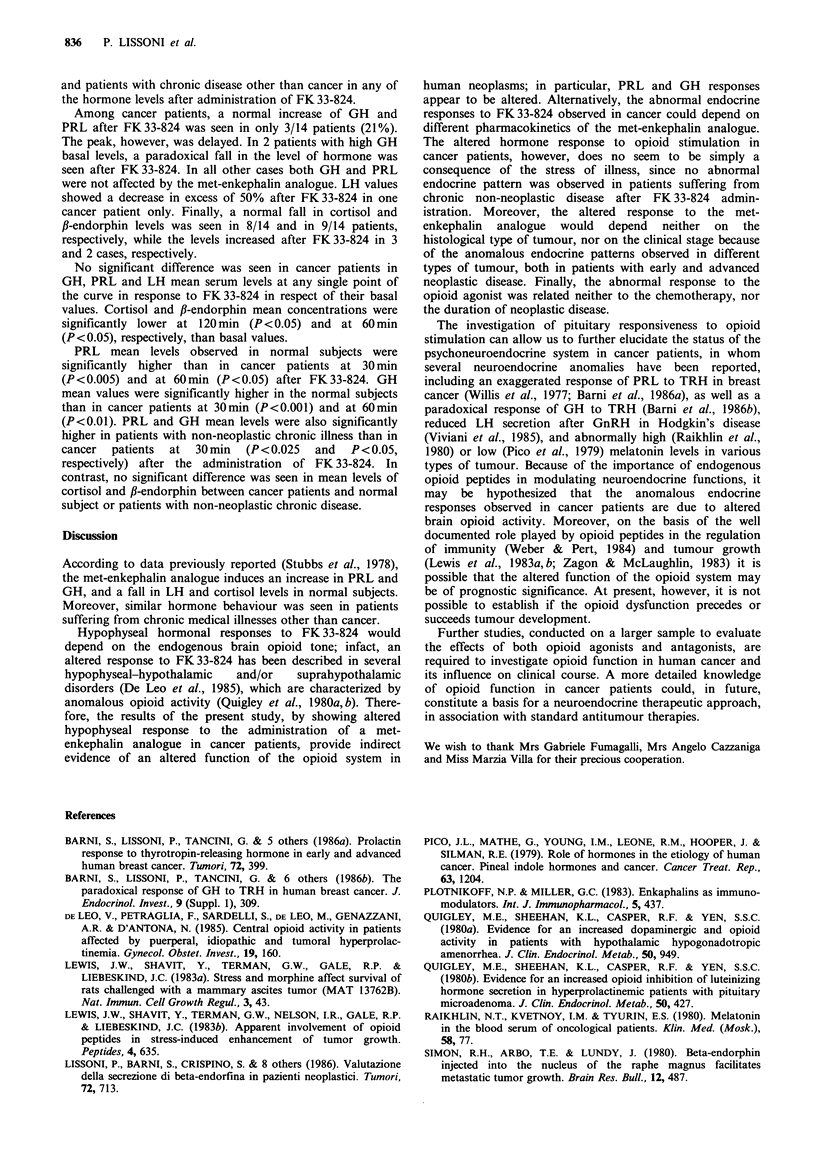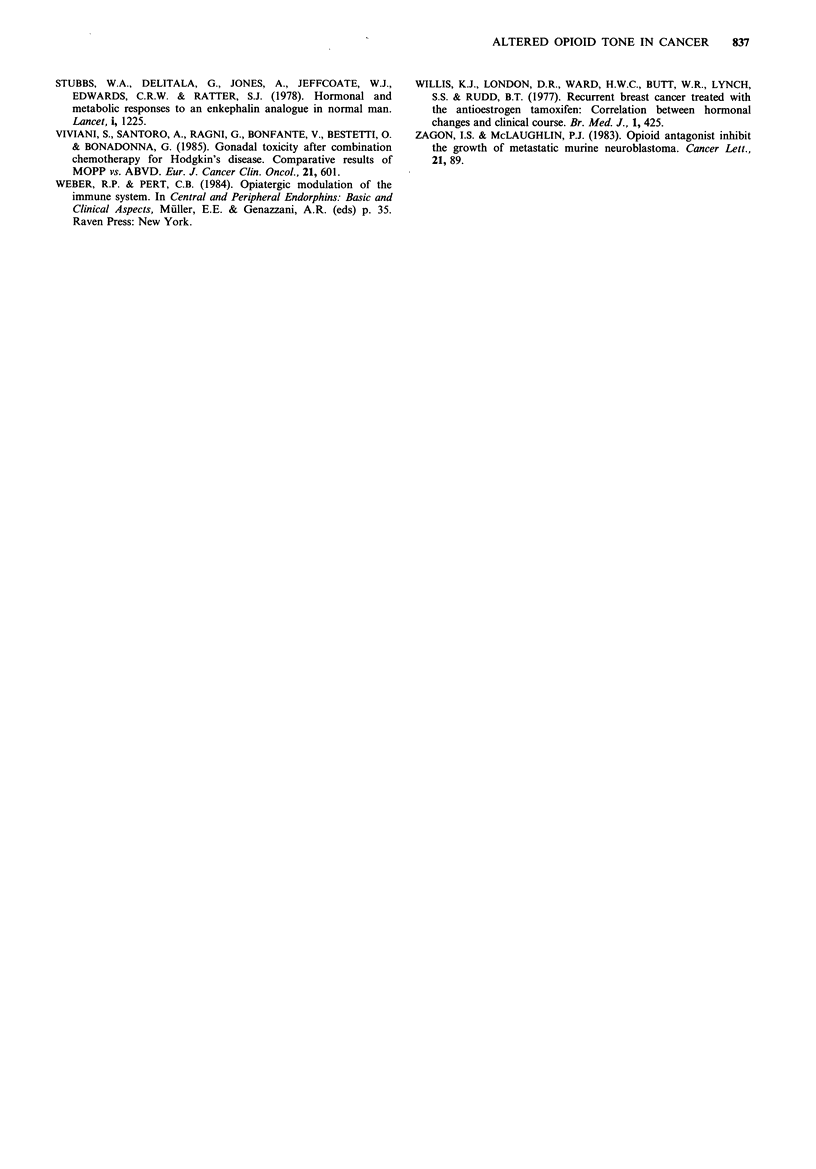# Evidence for altered opioid activity in patients with cancer.

**DOI:** 10.1038/bjc.1987.300

**Published:** 1987-12

**Authors:** P. Lissoni, S. Barni, F. Paolorossi, S. Crispino, F. Rovelli, L. Ferri, G. Delitala, G. Tancini

**Affiliations:** Division of Radiation Oncology, Ospedale San Gerardo, Monza, Milan, Italy.

## Abstract

Endogenous opioid peptides have been shown to be involved in the regulation of tumour growth. At present, however, no data are available about the secretion of opioid peptides in cancer patients. To draw some preliminary conclusions on opioid brain function in human neoplasms, we evaluated hypophyseal hormone responses to the administration of a met-enkephalin analogue, FK 33-824. The study included 14 patients affected by early or advanced neoplastic disease, 12 healthy subjects and 7 patients with a chronic medical illness other than cancer. FK 33-824 was given intravenously at a dose of 0.3 mg. Venous blood samples were collected at zero time, and 30, 60 and 120 min after drug administration. In each sample, PRL, GH, LH, cortisol and beta-endorphin levels were measured by RIA. In all normal subjects and in patients with non-neoplastic chronic illness, FK 33-824 induced a rise in PRL and GH levels, and a decrease in LH, cortisol and beta-endorphin. A normal endocrine response to FK 33-824 was seen in our cancer patient only, while in the other cases with tumour no hormonal changes or a paradoxical response were seen after FK 33-824. Based on the fact that an abnormal endocrine response to FK 33-824 has been described in hypothalamic-pituitary disorders, in which anomalous brain opioid activity has been demonstrated, these results suggest the existence of an altered function of the opioid system in cancer patients, the clinical importance of which remains to be determined.


*Br. J. Cancer* (1987) **56**, 834–837 © The Macmillan Press Ltd., 1987

### Evidence for altered opioid activity in patients with cancer

P. Lissoni[1], S. Barni[1], F. Paolorossi[1], S. Crispino[1], F. Rovelli[1], L. Ferri[1], G. Delitala[2] & G. Tancini[1]

[1]*Division of Radiation Oncology, Ospedale San Gerardo, Monza, Milan; and* [2]*Department of Endocrinology, University of Sassari, Sassari, Italy.*

**Summary** Endogenous opioid peptides have been shown to be involved in the regulation of tumour growth. At present, however, no data are available about the secretion of opioid peptides in cancer patients.

To draw some preliminary conclusions on opioid brain function in human neoplasms, we evaluated hypophyseal hormone responses to the administration of a met-enkephalin analogue, FK 33-824. The study included 14 patients affected by early or advanced neoplastic disease, 12 healthy subjects and 7 patients with a chronic medical illness other than cancer.

FK 33-824 was given intravenously at a dose of 0.3 mg. Venous blood samples were collected at zero time, and 30, 60 and 120 min after drug administration. In each sample, PRL, GH, LH, cortisol and $\beta$-endorphin levels were measured by RIA.

In all normal subjects and in patients with non-neoplastic chronic illness, FK 33-824 induced a rise in PRL and GH levels, and a decrease in LH, cortisol and $\beta$-endorphin. A normal endocrine response to FK 33-824 was seen in one cancer patient only, while in the other cases with tumour no hormonal changes or a paradoxical response were seen after FK 33-824.

Based on the fact that an abnormal endocrine response to FK 33-824 has been described in hypothalamic-pituitary disorders, in which anomalous brain opioid activity has been demonstrated, these results suggest the existence of an altered function of the opioid system in cancer patients, the clinical importance of which remains to be determined.

There is mounting evidence indicating that endogenous opiates play a critical part in the control of immune functions (Weber & Pert, 1984). Moreover, recent experimental observations seem to demonstrate an involvement of endogenous opioid peptides in the regulation of tumour growth. As regards this hypothesis, however, the results are contradictory, since opioid substances have been seen to exert either a stimulatory (Lewis *et al.*, 1983*a*, *b*; Simon *et al.*, 1984) or an inhibitory role (Plotnikoff & Miller, 1983) on tumour growth, depending on the different experimental conditions. Opioid antagonists may also have stimulatory and inhibitory effects, depending on the dosage (Zagon & McLaughlin, 1983).

As far as the evaluation of endogenous opioid secretion in cancer patients is concerned, no data are yet available. Preliminary results would seem to suggest an anomalous $\beta$-endorphin circadian rhythm in human neoplasms (Lissoni *et al.*, 1986), the clinical significance of which remains to be determined.

To further elucidate the nature of the opioid activity in human cancer, we studied the effects of a met-enkephalin analogue on the release of hypophyseal hormones in a group of patients suffering from early or advanced neoplastic disease.

### Materials and methods

The study was carried out on 14 patients of both sexes (5 men, 4 premenopausal and 5 postmenopausal women), aged between 32 and 53 years (mean age 46.4 years), with histologically proven neoplastic disease. Patients were followed in the outpatient clinic of San Gerardo Hospital, Monza. Diagnosis of cancer was made for at least 4 months prior to study (4 months–5 years; mean 3.8 years). Breast cancer and lung carcinoma were the two neoplasms most frequently represented in our cases. Chronic pain was present in 2 patients only. Clinical data of cancer patients are given in Table I.

Correspondence: P. Lissoni.
Received 31 March 1987; and in revised form, 10 July 1987.

As controls, 12 healthy volunteers (6 men, 4 premenopausal and 2 postmenopausal women) of same age (28–51 yrs; mean 42.4) were included in the study. Volunteers were from among hospital attendants. Moreover, a second group, consisting of 7 patients (4 men, 2 premenopausal and one postmenopausal women), aged between 31 and 58 years (mean 49.3), affected by a chronic medical illness other than cancer, was evaluated. None of the patients was hospitalized during the study. The experimental protocol was explained to patients and volunteers, and informed consent was obtained.

None of the cancer patients had been previously treated with oestrogens, antioestrogens or glucocorticoids. No antiemetics or other psychotropic drugs were given for at least one week prior to study. Moreover, no patient received opiates to relieve pain; finally, patients who received chemotherapy were observed for at least 20 days after the last administration of cytotoxic drugs.

All procedures were begun at 9.00 after an overnight fast during the summer season. FK 33-824 (DAMME; Sandoz, Basel, Switzerland), a longer-acting met-enkephalin analogue, was administered i.v. at a dose of 0.3 mg in 10 ml of saline solution over 5 min. Venous blood samples were drawn through an indwelling catheter at zero time, and 30, 60 and 120 min after FK 33-824 infusion. On a separate occasion and after an interval of at least one week, the healthy subjects were studied during a saline infusion only.

In each venous sample, serum levels of PRL, GH, LH, cortisol, and plasma concentrations of $\beta$-endorphin were measured. Sera and plasma were obtained by centrifugation, and stored at $-20$°C until assayed. Hormonal assays were made within 10 days after blood sampling. PRL, GH, LH and cortisol serum levels were measured by RIA using commercial available kits (Sclavo, Milan, Italy), while plasma values of $\beta$-endorphin were detected with the commercial kits developed by Nichols Institute Diagnostics (San Juan Capistrano, California). All samples were assayed in duplicate in a single assay. the intraassay and interassay coefficients of variation were below 6% and 9%, respectively.

Statistical analyses were performed by Student's $t$ test and analysis of variance according to the Newman Keuls test and

**Table I** Clinical data

| *Cases* | *Sex* | *Age (yrs)* | *Body weight (kg)* | *Tumour* | *TNM* | *Site of metastases* | *Performance status*[a] | *Chronic pain* | *Previous treatment*[b] |
|---|---|---|---|---|---|---|---|---|---|
| 1 | M | 51 | 77 | Squamous cell lung | $T_3N_2M_0$ | — | 70 | – | RT+CDDP |
| 2 | M | 48 | 82 | Squamous cell lung | $T_3N_2M_1$ | Brain | 90 | – | — |
| 3 | F | 46 | 69 | Squamous cell lung | $T_2N_2M_0$ | — | 80 | – | RT+CDDP−$VP_{16}$ |
| 4 | M | 53 | 71 | Small cell lung | $T_3N_2M_0$ | — | 60 | – | RT+CDD−$VP_{16}$ |
| 5 | F | 50 | 68 | Small cell lung | $T_3N_2M_1$ | Nodes, bone | 40 | + | CEV |
| 6 | F | 46 | 79 | Breast | $T \times N \times M_1$ | Bone | 60 | – | Surgery |
| 7 | F | 32 | 49 | Breast | $T_4N_3M_1$ | Liver | 20 | – | FEC |
| 8 | F | 48 | 67 | Breast | $T_2N_1M_1$ | Skin, bone, lung | 40 | + | FEC |
| 9 | F | 42 | 63 | Breast | $T_2N_1M_0$ | — | 100 | – | Surgery+CMF |
| 10 | F | 38 | 59 | Breast | $T_2N_1M_0$ | — | 100 | – | Surgery+CMF |
| 11 | M | 51 | 68 | Gastric | $T \times N \times M_1$ | Liver | 70 | – | Surgery+5-FU |
| 12 | M | 53 | 73 | Thymoma | — | — | 80 | – | — |
| 13 | F | 42 | 62 | Uterine cervix | $T_3N_1M_0$ | — | 100 | – | CDDP |
| 14 | F | 49 | 83 | Uterine cervix | $T_3N_1M_0$ | — | 90 | – | CDDP |

[a]Karnofsky; [b]RT: Radiotherapy; CDDP: *Cis*-platinum; $VP_{16}$: Etoposide; CEV: Cyclophosphamide, Epirubicin, Vincristine; FEC: Fluorouracil, Epirubicin, Cyclophosphamide; CMF: Cyclophosphamide, Methotrexate, Fluorouracil; 5-FU: Fluorouracil.

adjusted for a correction factor. Results were reported as the mean ± s.e. Hormonal basal levels were considered as 'high' or 'low' when they were greater or less than 2 s.d. relative to those observed in healthy subjects.

### Results

All patients and healthy subjects experienced an unpleasant feeling of heaviness of the body, particularly in the legs, of a few minutes' duration only, associated with a facial flushing and headache in some cases. No change in pulse or blood pressure was observed.

Figures 1 and 2 illustrate blood levels (mean ± s.e.) of PRL and GH, respectively, observed in healthy subjects, cancer patients and those with chronic illness other than cancer.

In all healthy volunteers and in patients with chronic disease other than cancer, increases of PRL (>200%) and of GH (>10 pg ml$^{-1}$) were seen after FK 33-824, with a peak at 30 min. LH, cortisol and β-endorphin decreased after FK 33-824, with a fall >50% in respect to their basal values. The lowest levels of LH and β-endorphin were found at 60 min and those of cortisol after 120 min. In the healthy volunteers, PRL and GH serum mean levels observed at 30 and 60 min after FK 33-824 were significantly higher than both those seen during saline infusion at the same times and those found in basal conditions ($P<0.001$). Cortisol and β-endorphin mean values were significantly lower ($P<0.005$) after FK 33-824 than during saline at 120 and 60 min, respectively. Moreover, LH mean values were significantly lower at 30, 60 and 120 min after FK 33-824 than during saline and at their basal levels ($P<0.001$). Finally, no significant differences were seen between healthy subjects

**Figure 1** PRL serum mean levels after FK 33-824 in healthy subjects (●——●; $n=12$) and in patients with neoplastic (△——△; $n=14$) and non-neoplastic (○——○; $n=7$) disease.

**Figure 2** GH serum mean levels after FK 33-824 in healthy subjects (●——●; $n=12$) and in patients with neoplastic (△——△; $n=14$) and non-neoplastic (○—○; $n=7$) disease.

and patients with chronic disease other than cancer in any of the hormone levels after administration of FK 33-824.

Among cancer patients, a normal increase of GH and PRL after FK 33-824 was seen in only 3/14 patients (21%). The peak, however, was delayed. In 2 patients with high GH basal levels, a paradoxical fall in the level of hormone was seen after FK 33-824. In all other cases both GH and PRL were not affected by the met-enkephalin analogue. LH values showed a decrease in excess of 50% after FK 33-824 in one cancer patient only. Finally, a normal fall in cortisol and $\beta$-endorphin levels was seen in 8/14 and in 9/14 patients, respectively, while the levels increased after FK 33-824 in 3 and 2 cases, respectively.

No significant difference was seen in cancer patients in GH, PRL and LH mean serum levels at any single point of the curve in response to FK 33-824 in respect of their basal values. Cortisol and $\beta$-endorphin mean concentrations were significantly lower at 120 min ($P<0.05$) and at 60 min ($P<0.05$), respectively, than basal values.

PRL mean levels observed in normal subjects were significantly higher than in cancer patients at 30 min ($P<0.005$) and at 60 min ($P<0.05$) after FK 33-824. GH mean values were significantly higher in the normal subjects than in cancer patients at 30 min ($P<0.001$) and at 60 min ($P<0.01$). PRL and GH mean levels were also significantly higher in patients with non-neoplastic chronic illness than in cancer patients at 30 min ($P<0.025$ and $P<0.05$, respectively) after the administration of FK 33-824. In contrast, no significant difference was seen in mean levels of cortisol and $\beta$-endorphin between cancer patients and normal subject or patients with non-neoplastic chronic disease.

### Discussion

According to data previously reported (Stubbs *et al.*, 1978), the met-enkephalin analogue induces an increase in PRL and GH, and a fall in LH and cortisol levels in normal subjects. Moreover, similar hormone behaviour was seen in patients suffering from chronic medical illnesses other than cancer.

Hypophyseal hormonal responses to FK 33-824 would depend on the endogenous brain opioid tone; infact, an altered response to FK 33-824 has been described in several hypophyseal–hypothalamic and/or suprahypothalamic disorders (De Leo *et al.*, 1985), which are characterized by anomalous opioid activity (Quigley *et al.*, 1980*a*, *b*). Therefore, the results of the present study, by showing altered hypophyseal response to the administration of a met-enkephalin analogue in cancer patients, provide indirect evidence of an altered function of the opioid system in human neoplasms; in particular, PRL and GH responses appear to be altered. Alternatively, the abnormal endocrine responses to FK 33-824 observed in cancer could depend on different pharmacokinetics of the met-enkephalin analogue. The altered hormone response to opioid stimulation in cancer patients, however, does no seem to be simply a consequence of the stress of illness, since no abnormal endocrine pattern was observed in patients suffering from chronic non-neoplastic disease after FK 33-824 administration. Moreover, the altered response to the met-enkephalin analogue would depend neither on the histological type of tumour, nor on the clinical stage because of the anomalous endocrine patterns observed in different types of tumour, both in patients with early and advanced neoplastic disease. Finally, the abnormal response to the opioid agonist was related neither to the chemotherapy, nor the duration of neoplastic disease.

The investigation of pituitary responsiveness to opioid stimulation can allow us to further elucidate the status of the psychoneuroendocrine system in cancer patients, in whom several neuroendocrine anomalies have been reported, including an exaggerated response of PRL to TRH in breast cancer (Willis *et al.*, 1977; Barni *et al.*, 1986*a*), as well as a paradoxical response of GH to TRH (Barni *et al.*, 1986*b*), reduced LH secretion after GnRH in Hodgkin's disease (Viviani *et al.*, 1985), and abnormally high (Raikhlin *et al.*, 1980) or low (Pico *et al.*, 1979) melatonin levels in various types of tumour. Because of the importance of endogenous opioid peptides in modulating neuroendocrine functions, it may be hypothesized that the anomalous endocrine responses observed in cancer patients are due to altered brain opioid activity. Moreover, on the basis of the well documented role played by opioid peptides in the regulation of immunity (Weber & Pert, 1984) and tumour growth (Lewis *et al.*, 1983*a*, *b*; Zagon & McLaughlin, 1983) it is possible that the altered function of the opioid system may be of prognostic significance. At present, however, it is not possible to establish if the opioid dysfunction precedes or succeeds tumour development.

Further studies, conducted on a larger sample to evaluate the effects of both opioid agonists and antagonists, are required to investigate opioid function in human cancer and its influence on clinical course. A more detailed knowledge of opioid function in cancer patients could, in future, constitute a basis for a neuroendocrine therapeutic approach, in association with standard antitumour therapies.

We wish to thank Mrs Gabriele Fumagalli, Mrs Angelo Cazzaniga and Miss Marzia Villa for their precious cooperation.

### References

BARNI, S., LISSONI, P., TANCINI, G. & 5 others (1986*a*). Prolactin response to thyrotropin-releasing hormone in early and advanced human breast cancer. *Tumori*, **72**, 399.

BARNI, S., LISSONI, P., TANCINI, G. & 6 others (1986*b*). The paradoxical response of GH to TRH in human breast cancer. *J. Endocrinol. Invest.*, **9** (Suppl. 1), 309.

DE LEO, V., PETRAGLIA, F., SARDELLI, S., DE LEO, M., GENAZZANI, A.R. & D'ANTONA, N. (1985). Central opioid activity in patients affected by puerperal, idiopathic and tumoral hyperprolactinemia. *Gynecol. Obstet. Invest.*, **19**, 160.

LEWIS, J.W., SHAVIT, Y., TERMAN, G.W., GALE, R.P. & LIEBESKIND, J.C. (1983*a*). Stress and morphine affect survival of rats challenged with a mammary ascites tumor (MAT 13762B). *Nat. Immun. Cell Growth Regul.*, **3**, 43.

LEWIS, J.W., SHAVIT, Y., TERMAN, G.W., NELSON, I.R., GALE, R.P. & LIEBESKIND, J.C. (1983*b*). Apparent involvement of opioid peptides in stress-induced enhancement of tumor growth. *Peptides*, **4**, 635.

LISSONI, P., BARNI, S., CRISPINO, S. & 8 others (1986). Valutazione della secrezione di beta-endorfina in pazienti neoplastici. *Tumori*, **72**, 713.

PICO, J.L., MATHE, G., YOUNG, I.M., LEONE, R.M., HOOPER, J. & SILMAN, R.E. (1979). Role of hormones in the etiology of human cancer. Pineal indole hormones and cancer. *Cancer Treat. Rep.*, **63**, 1204.

PLOTNIKOFF, N.P. & MILLER, G.C. (1983). Enkaphalins as immunomodulators. *Int. J. Immunopharmacol.*, **5**, 437.

QUIGLEY, M.E., SHEEHAN, K.L., CASPER, R.F. & YEN, S.S.C. (1980*a*). Evidence for an increased dopaminergic and opioid activity in patients with hypothalamic hypogonadotropic amenorrhea. *J. Clin. Endocrinol. Metab.*, **50**, 949.

QUIGLEY, M.E., SHEEHAN, K.L., CASPER, R.F. & YEN, S.S.C. (1980*b*). Evidence for an increased opioid inhibition of luteinizing hormone secretion in hyperprolactinemic patients with pituitary microadenoma. *J. Clin. Endocrinol. Metab.*, **50**, 427.

RAIKHLIN, N.T., KVETNOY, I.M. & TYURIN, E.S. (1980). Melatonin in the blood serum of oncological patients. *Klin. Med. (Mosk.)*, **58**, 77.

SIMON, R.H., ARBO, T.E. & LUNDY, J. (1980). Beta-endorphin injected into the nucleus of the raphe magnus facilitates metastatic tumor growth. *Brain Res. Bull.*, **12**, 487.

STUBBS, W.A., DELITALA, G., JONES, A., JEFFCOATE, W.J., EDWARDS, C.R.W. & RATTER, S.J. (1978). Hormonal and metabolic responses to an enkephalin analogue in normal man. *Lancet*, **i**, 1225.

VIVIANI, S., SANTORO, A., RAGNI, G., BONFANTE, V., BESTETTI, O. & BONADONNA, G. (1985). Gonadal toxicity after combination chemotherapy for Hodgkin's disease. Comparative results of MOPP *vs*. ABVD. *Eur. J. Cancer Clin. Oncol.*, **21**, 601.

WEBER, R.P. & PERT, C.B. (1984). Opiatergic modulation of the immune system. In *Central and Peripheral Endorphins: Basic and Clinical Aspects*, Müller, E.E. & Genazzani, A.R. (eds) p. 35. Raven Press: New York.

WILLIS, K.J., LONDON, D.R., WARD, H.W.C., BUTT, W.R., LYNCH, S.S. & RUDD, B.T. (1977). Recurrent breast cancer treated with the antioestrogen tamoxifen: Correlation between hormonal changes and clinical course. *Br. Med. J.*, **1**, 425.

ZAGON, I.S. & McLAUGHLIN, P.J. (1983). Opioid antagonist inhibit the growth of metastatic murine neuroblastoma. *Cancer Lett.*, **21**, 89.